# Low-Alkali Assisted Instant Catapult Steam Explosion Enhances Enzymatic Hydrolysis of Corn Stover by Alleviating Anti-Enzymatic Hydrolysis Factors Through Lignin Structural Rearrangement

**DOI:** 10.3390/polym17233148

**Published:** 2025-11-26

**Authors:** Hongsen Zhang, Wenjing Li, Yufei Yang, Guotao Mao, Fengqin Wang, Hui Xie, Zengping Su, Jingliang Xu, Andong Song

**Affiliations:** 1 College of Life Sciences, Henan Agricultural University, No. 15 Longzihu University Campus, Zhengzhou 450046, China; hszhang@henau.edu.cn (H.Z.); lwj@stu.henau.edu.cn (W.L.); yyf@stu.henau.edu.cn (Y.Y.); w_fengqin@henau.edu.cn (F.W.); xiehui@henau.edu.cn (H.X.); suzengping@henau.edu.cn (Z.S.); 2 School of Chemical Engineering, Zhengzhou University, No. 100 Science Avenue, Zhengzhou 450001, China; xujl@zzu.edu.cn

**Keywords:** low-alkali, instant catapult steam explosion, corn stover, enzymatic hydrolysis, anti-enzymatic hydrolysis factors

## Abstract

The effective deconstruction of lignocellulosic biomass is essential for sustainable biorefineries. In this study, corn stover was pretreated by low-alkali (1–5 wt% NaOH) pre-impregnation assisted instant catapult steam explosion (ICSE) to investigate its influence on enzymatic hydrolysis efficiency and the mechanism of lignin-derived anti-enzymatic factors. The results showed that this pretreatment effectively enhanced glucose yield. Under 4–5% NaOH conditions, washed samples achieved glucose yields above 98%. At 4% NaOH, the glucose yields of washed and unwashed groups were 98.88% and 56.34%, respectively, indicating that washing removed soluble inhibitors. LC-MS analysis identified three major water-soluble inhibitory compounds-vanillin, syringaldehyde, and 2-carboxybenzaldehyde-confirming their negative effects on cellulase activity. The alkali-soluble lignin content of unwashed samples (43.28%) was 1.36 times higher than that of washed samples (31.93%), demonstrating its role as a water-insoluble inhibitory factor. Moreover, SEM, XRD, FTIR, and contact angle analyses revealed that 5% NaOH treatment enhanced lignin solubilization, induced structural rearrangement and interfacial hydrophilic reconstruction, and increased cellulose crystallinity and enzyme accessibility. These findings elucidate the mechanistic pathways of lignin transformation and inhibition mitigation, providing valuable insights for efficient and sustainable biomass conversion.

## 1. Introduction

With the growth of global energy demand and the depletion of fossil fuel resources, the development of renewable biomass energy has become an important way to solve the energy crisis and environmental pollution [1]. Lignocellulose, as the most abundant renewable carbon source on Earth, has an annual output of about 200 billion tons, and its efficient conversion technology is a core challenge in the field of biorefining [2]. As a large agricultural country, China has extremely rich corn stover resources. However, due to its complex lignocellulose structure, its efficient conversion and utilization are severely limited [3,4,5]. Lignocellulose is mainly composed of cellulose, hemicellulose, and lignin. In the natural state, they are intertwined to form a dense structure, hindering the contact between enzyme molecules and cellulose [6], lignin covers the surface of cellulose through hydrophobic interactions.

Currently, pretreatment methods include physical methods (mechanical comminution, steam explosion, et al.), chemical methods (acid/alkali treatment, oxidation treatment, et al.), and biological methods (degradation by white rot fungi) [7,8,9]. Steam explosion technology, as an effective physical pretreatment method, has been widely applied in multiple fields, such as biorefinery, food and feed, et al. [10]. It can disrupt the structure of lignocellulose and increase its accessible surface area by releasing pressure instantaneously under high temperature and pressure conditions. However, the high temperature and pressure conditions may lead to the degradation of sugars, generating inhibitors such as furfural and 5-hydroxymethylfurfural (HMF), which can affect the activity of subsequent fermentation microorganisms [11]. The instant catapult steam explosion (ICSE) was used in this study, by simulating the fracture of a pressure vessel, is designed with the deflation cross-sectional area equal to that of the pressure cavity. The explosion time is only 0.0875 s, which maximized the instantaneity of the explosion [12].

Alkali pretreatment can effectively improve enzyme accessibility by saponifying ester bonds and swelling cellulose [13]. Traditional high-concentration alkali (12 wt%, 12 g NaOH per 100 g dry biomass) treatment can achieve a lignin removal rate of 60–80% [14]. However, the use of high alkali loadings also brings substantial environmental burdens. Conventional systems typically rely on 12–15 wt% NaOH, corresponding to chemical inputs of approximately 120–150 g·kg^−1^ biomass, and generate wastewater with COD values frequently exceeding 20,000–30,000 mg·L^−1^, which increases treatment cost by 30–50% [15,16]. In addition, the harsh alkaline environment accelerates cellulose degradation, converting approximately 15–20% of cellulose into non-fermentable oligosaccharides [17], and promotes extensive lignin depolymerization that produces large quantities of phenolic inhibitors. By contrast, the low-alkali ICSE configuration evaluated in this work operates at only 5 wt% NaOH. Based on stoichiometric alkali use and the minimal rinsing required during ICSE, the total alkali input can be reasonably estimated to be around 50 g·kg^−1^ biomass. The milder pretreatment conditions also suggest markedly lower COD levels, likely below 10,000 mg·L^−1^, while water demand is expected to be substantially reduced compared with the 15–20 m^3^·t^−1^ required in conventional high-alkali processes [18]. These quantitative differences highlight the potential of low-alkali ICSE to reduce chemical consumption, wastewater load, and overall environmental discharge, offering a more sustainable alternative while maintaining effective structural disruption.

In recent years, several studies have attempted to reduce NaOH dosage below 5 wt% to lower chemical input and environmental burden. For example, Li et al. applied 1–3 wt% NaOH combined with wet ball milling and achieved high glucose yields, but the process required additional mechanical energy, limiting its scalability [19]. Similarly, Hu et al. reported efficient saccharification with 1 wt% NaOH coupled with alkaline H_2_O_2_/UV treatment, yet this approach depended on oxidants and prolonged reaction times [20]. These studies highlight that while low-alkali pretreatments are promising, their reliance on external energy, additives, or complex chemistries remains a barrier to practical application.

To address these challenges, the present work introduces a low-alkali (1–5 wt% NaOH) pretreatment coupled with instant catapult steam explosion (ICSE). The synergy between alkali impregnation and explosive depressurization enhances lignin cleavage and fiber disruption even at low NaOH levels. Compared with conventional high-alkali pretreatment or other low-alkali approaches, this strategy reduces chemical consumption, minimizes washing demand, and avoids reliance on oxidants or energy-intensive processes.

Another important contribution of this work is the mechanistic investigation of lignin-derived inhibitors under low-alkali ICSE conditions. Although structural modification of lignocellulose is achieved, free lignin generated during explosion can redeposit on cellulose surfaces after cooling, forming physical barriers that reduce enzymatic accessibility. Prior studies have shown that lignin recondensation may increase enzyme dosage requirements by 15–20% [21]. Phenolic and aldehyde derivatives generated during the pretreatment process may also inhibit the activity of cellulase, leading to a decrease in enzymatic hydrolysis efficiency [22]. Bhatia et al. [23] found that water-soluble phenolic substances and aromatic compounds generated during the pretreatment process can inhibit enzyme activity, Saini et al. [24] showed that non-productive enzyme-lignin binding can lead to a decrease in enzyme activity. Other studies have indicated that the contact angle value of lignin is higher than that of cellulose, indicating that lignin has stronger hydrophobicity and cellulase tends to adsorb lignin preferentially [25]. In addition to the non-productive adsorption of enzymes, the inhibitory effect of lignin on the enzymatic hydrolysis of cellulose is also reflected in the steric hindrance effect. During the pretreatment stage of lignocellulosic raw materials, lignin can form a barrier through two pathways: first, it migrates to the fiber surface, causing the redistribution of the biomass matrix; second, the dissolved lignin condenses into droplets and deposits on the surface of cellulose, significantly hindering the adsorption process of cellulase and thus inhibiting the effective progress of the enzymatic hydrolysis reaction [26].

This study aims to investigate the elimination effects of anti-enzymatic hydrolysis factors in corn stover by varying concentrations of NaOH assisted ICSE. By systematically analyzing the enzymatic hydrolysis performance before and after water washing, as well as following the reintroduction of key inhibitors, and through comprehensive physicochemical characterizations, the underlying mechanisms of enzymatic inhibition are elucidated. The findings not only provide critical insights into pretreatment optimization and enzyme accessibility but also pave the way for the high-value utilization of corn stover. Ultimately, this work offers a significant contribution to the advancement of the biorefinery industry by enabling more efficient, cleaner, and economically viable biomass conversion technologies.

## 2. Materials and Methods

### 2.1. Materials

The corn stover was harvested in October 2022 when it was ripe, collected from Donghai County, Lianyungang City, Jiangsu Province. The corn stover was crushed and then passed through a 60-mesh sieve. After that, it was washed with deionized water and dried at 65 °C until reaching a constant mass. The dried corn stover was stored at room temperature (25 °C) for future use. The cellulase (CTec2) was purchased from Novozymes Biotech Co., Ltd., Tianjin, China, with an enzyme activity of 100 FPU/mL. Sodium hydroxide (NaOH) was purchased from Shanghai Macklin Biochemical Co., Ltd., Shanghai, China.

### 2.2. Pretreatment of Corn Stover

Firstly, 180 g of the dried corn stover was weighed and placed in a glass beaker. Subsequently, it was added into a mixed solution containing NaOH accounting for 1–5 wt% of the dry weight of the corn stover and distilled water with a solid-liquid mass ratio of corn stover to water being 1:1, and the mixture was stirred evenly.

The corn stover soaked with different concentrations of NaOH was used for ICSE. The ICSE pressure was 2.0 MPa, and the retention time was 360 s. (The ICSE machine used in this study was purchased from Henan Hebi Zhengdao Enterprise Co., Ltd., Hebi, China)

Half of the exploded materials were taken out and directly dried, and the remaining materials were replenished with water to a ratio of 1:20. Then, they were placed in a water bath at 60 °C and left to stand for 1 h, with stirring every 10 min. After that, a circulating water vacuum pump was used for suction filtration to separate the solid from the liquid. After collecting the washing liquid, the materials were rinsed thoroughly with deionized water.

### 2.3. Determination Methods of Components in Lignocellulosic Raw Materials

After the corn stover was pretreated according to the national standardized method, the chemical composition of the sample was analyzed using the method developed by the National Renewable Energy Laboratory (NREL) of the United States [27]. The polymeric carbohydrates were hydrolyzed into monomeric sugar forms, and all monomeric sugars were measured by High Performance Liquid Chromatography (HPLC) was sourced from Shimadzu Corporation, Tokyo, Japan. The lignin content was determined based on the water loss of the material. The percentage concentration of the polymer was calculated according to the concentration of the corresponding monomeric sugars. The conversion coefficients for xylose and arabinose were 0.88, and the conversion coefficient for glucose was 0.90.

### 2.4. Enzymatic Hydrolysis Conditions and Determination Method of Glucose

Weigh 0.5 g of dry matter and add it to 20 mL of sodium citrate buffer (0.05 M, pH 4.8). Mix them in a 50-mL Erlenmeyer flask. Meanwhile, add 10 g/L tetracycline hydrochloride and 150 μL of cellulase (15 FPU/g corn stover). React the above sample at 50 °C and 180 rpm for 72 h. Take samples every 24 h. Transfer 1 mL of the sample to a centrifuge and centrifuge it at 12,000 rpm for 10 min. Take the supernatant and determine the sugar content in the liquid by HPLC.

Determination of Glucose: Glucose was determined by HPLC (LC-20A, refractive index detector RID-10A, Shimadzu, Kyoto, Japan) with Aminex HPX-87H column (Bio-Rad, Hercules, CA, USA). The mobile phase was 0.005 mM H_2_SO_4_ with a flow rate of 0.6 mL/min, and the column temperature was set at 65 °C [28]. The sugar yield was calculated according to Equation (1):

y_g_ = (m_1_ × 0.9)/m_2_ × 100%, (1)

In the formula: y_g_ is the glucose yield (%); m_1_ is the amount of glucose in the hydrolysate (g); m_2_ is the cellulose content in the sample (g); 0.9 is the conversion coefficient for converting glucose to glucan.

### 2.5. Extraction and Analysis Methods of Water-Soluble Anti-Enzymatic Hydrolysis Factors

The washing liquid of corn stover after pretreatment and then washed with water at a ratio of 1:20 was taken for analysis. Before the analysis, a method similar to that reported by Chen et al. [29] was used to extract the target analytes from the hydrolysate with methyl tert-butyl ether (MTBE). 5 mL of the collected washing liquid was pipetted and added to 45 mL of MTBE solution. The mixture was placed in a shaker at 25 °C with a rotation speed of 180 rpm and shaken for 15 min. After separating the two phases by a centrifuge, the aqueous layer was extracted for a second time to maximize the recovery rate of the washing liquid. The volumes of the two MTBE extracts were combined, and the combined extract was rotary-evaporated to 1–2 mL, then diluted to 5 mL with water. Subsequently, the diluted extract was detected in the negative ion mode using an AB SCIEX Triple TOF 6600+ high-resolution liquid chromatography-mass spectrometry equipped with SCIEX OS data processing software. The chromatographic column was a Phenomenex 2.6 um XB-C18 (100 × 2.1 mm, Phenomenex, Torrance, CA, USA). The scanning mode was set as ISVF: 5500 V; CE: 35 ± 15 V, and the ion source temperature was 550 °C. After the test, the results were matched with the database.

The phenolic inhibitors (vanillin, syringaldehyde, and 2-carboxybenzaldehyde) were quantitatively analyzed by ultra-high-performance liquid chromatography An ultraviolet (UV) detector (Model: DVD-3000, Thermo Fisher Scientific Inc., Waltham, MA, USA) was used, with a chromatographic column (Agilent C18 column). The column temperature was set at 35 °C, and the detection wavelength was 280 nm. The initial mobile phase consisted of 0.1% formic acid and 10% acetonitrile at a volume ratio of 9:1. Gradient elution was adopted, with the specific settings as follows: acetonitrile concentration was increased from 0% to 10% within 1–15 min, then raised from 10% to 35% during 15–20 min, and finally maintained at 10% from 20–30 min. The analysis was performed at a flow rate of 1.0 mL/min.

Preparation of Standard Curves: Stock solutions of vanillin, syringaldehyde, and 2-carboxybenzaldehyde were accurately prepared at a concentration of 1.0 mg/mL, respectively. These stock solutions were serially diluted to obtain standard solutions with concentrations of 0.2, 0.4, 0.6, 0.8, and 1.0 mg/L. Using concentration as the abscissa (*x*-axis) and peak area as the ordinate (*y*-axis), linear regression analysis was performed with Excel. The resulting standard curves were as follows: vanillin: y = 2960.1x + 11,980 (R^2^ = 0.9952); syringaldehyde: y = 15659x + 688.7 (R^2^ = 0.995); 2-carboxybenzaldehyde: y = 3275.7x + 24.789 (R^2^ = 0.9978).

### 2.6. Quantitative Analysis Methods for Vanillin, 2-Carboxybenzaldehyde, and Syringaldehyde in Corn Stover Hydrolysate

The analysis was performed using a Thermo Fisher Scientific UV detector (Model: DVD-3000, Thermo Fisher Scientific Inc., Waltham, MA, USA) equipped with an Agilent C18 column maintained at 35 °C. Detection was carried out at a wavelength of 280 nm. The initial mobile phase consisted of 0.1% formic acid and 10% acetonitrile in a 9:1 ratio. A gradient elution program was employed as follows: acetonitrile concentration was increased from 0% to 10% over 1–15 min, further increased from 10% to 35% from 15–20 min, and finally held at 10% from 20–30 min. The flow rate was maintained at 1.0 mL/min throughout the analysis.

### 2.7. Extraction Method of Alkali-Soluble Lignin Solid

Accurately weigh 5 g of 4% NaOH-assisted ICSE pretreated and then water-washed corn stover, as well as ICSE pretreated but unwashed corn stover, both of which have passed through a 60-mesh sieve. Add them separately to a 40 g/L NaOH solution at a solid-to-liquid ratio of 1:30 (g/mL). Place the mixtures in a water bath at 60 °C for 2.5 h. Then, perform suction filtration using a G3 crucible to remove the lignin dissolved in the filtrate and collect the filtrate. Adjust the pH of the filtrate to 1.5 with hydrochloric acid solution to precipitate the lignin. Let it stand overnight at room temperature until obvious stratification occurs. Remove the supernatant, then wash and centrifuge the precipitate first with a hydrochloric acid solution at pH 2 and then with distilled water. Freeze-dry the precipitate to obtain alkali-soluble lignin [30]. (The BTP-8ZL00X vacuum freeze-dryer was purchased from SP Scientific, Warminster, PA, USA).

### 2.8. Anti-Enzymatic Hydrolysis Factor Backfill Test Method

After the corn stover pretreated with 4% NaOH assisted ICSE underwent subsequent water-washed and unwashed treatments followed by enzymatic hydrolysis, a significant difference in hydrolysis efficiency was observed. Two main influencing factors were considered: first, the presence of water-soluble inhibitors in the washing solution; second, the existence of residual, undissolved lignin in both washed and unwashed samples. To further investigate their effects, the control group was established using the washed corn stover pretreated with 4% NaOH assisted ICSE, and various anti-enzymatic hydrolysis factors were subsequently supplemented.

#### 2.8.1. Lignin-Derived Water-Soluble Inhibitors Backfill Test Method

The concentrations of vanillin, syringaldehyde, and 2-carboxybenzaldehyde in the corn stover hydrolysate (4% NaOH assisted ICSE, unwashed) were determined to be 0.73 mg/mL, 0.25 mg/mL, and 0.27 mg/mL, respectively. Meanwhile, the three inhibitors were not detected in the corn stover hydrolysate (4% NaOH assisted ICSE, washed). According to these measured concentrations, inhibitor standards were backfilled into the enzymatic hydrolysis system (4% NaOH assisted ICSE, washed) at the following gradient concentrations:

(1) Vanillin: 0.25 mg/mL, 0.75 mg/mL, 2.5 mg/mL;
(2) Syringaldehyde: 0.25 mg/mL, 0.75 mg/mL, 1.5 mg/mL;
(3) 2-Carboxybenzaldehyde: 0.25 mg/mL, 0.75 mg/mL, 1.5 mg/mL.

Each treatment was conducted in triplicate, resulting in a total of 27 treatment groups. The enzymatic hydrolysis conditions and the method for calculating glucose yield were the same as described in Section 2.4.

#### 2.8.2. Alkali-Soluble Lignin Solid Backfill Test Method

The material used was corn stover (washed) that had been pretreated with 4% NaOH assisted ICSE. Alkali-soluble lignin, extracted from unwashed corn stover pretreated under the same conditions, was backfilled into the enzymatic hydrolysis system (4% NaOH assisted ICSE, washed). The amounts of alkali-soluble lignin added were 0.05 g, 0.075 g, and 0.1 g, respectively. Each treatment group was performed in triplicate. The enzymatic hydrolysis conditions and the method for calculating glucose yield were consistent with those described in Section 2.4.

### 2.9. Determination Method of Water-Soluble Lignin

Accurately weigh 0.5 g of the pretreated sample and add 20 mL of sodium citrate buffer. Incubate the mixture at 50 °C and 180 rpm for 24 h. After the reaction, centrifuge the mixture at 12,000 rpm for 10 min. Filter the resulting supernatant through a 0.22 μm filter membrane. Transfer the filtrate into a dialysis bag and perform dialysis against deionized water to remove interfering small-molecule sugars. Freeze-dry the dialyzed solution, then redissolve the lyophilized sample in 10 mL of sodium citrate buffer. Measure the absorbance (OD) of the solution at a detection wavelength of 320 nm using a UV-visible spectrophotometer to determine the content of water-soluble lignin.

Water-soluble lignin (mg/g) = (C × V × n)/m,(2)

In the formula: C is the lignin concentration (μg/mL) obtained from the standard curve, V is the final volume of the solution (mL), n is the dilution factor, m is the dry weight of the sample (g).

### 2.10. Analysis Method of Scanning Electron Microscope (SEM)

Accurately weigh 0.1 mg of the sample. After fixation, perform gold sputter-coating to prepare the sample for observation. Examine the microstructural features using a scanning electron microscope (S-4800, Hitachi, Japan) at an accelerating voltage of 15 kV [31].

### 2.11. Analysis Method of X-Ray Diffraction (XRD)

Accurately weigh 0.1 g of the sample and prepare it for XRD analysis. Measurements were carried out using an X-ray diffractometer (TD-3500, Dandong Tongda Technology Co., Ltd., Dandong, China) with Cu Kα radiation (λ = 1.5406 Å), operated at 40 kV and 40 mA. Scanning was performed over a 2θ range of 5° to 50°, with a step size of 0.02° and a scanning speed of 6°/min [32]. Crystallinity (CrI) and crystalline fraction were calculated using the following Equations:

CrI (%) = (*I*_002_ − *I_am_*)/*I*_002_ × 100%, (3)

Crystalline portion (%) = *I*_002_/(*I*_002_ + *I_am_*) × 100%, (4)

In the formula: CrI represents the X-ray crystallinity (%); Crystalline portion represents the X-ray crystallinity index (%); *I*_002_ is the maximum diffraction intensity of the lattice, that is, 2θ ≈ 22.3°; *I_am_* is the scattering intensity of the background diffraction in the amorphous region, that is, 2θ ≈ 16.15°.

### 2.12. Analysis Method of Infrared Spectroscopy

The dried sample was thoroughly mixed with potassium bromide (KBr) at a ratio of 1:100 and finely ground. The mixture was pressed into a pellet and scanned using a Fourier transform infrared (FT-IR) spectrometer (Nicolet iS10, Thermo Fisher Scientific Inc., Waltham, MA, USA) in the wavenumber range of 4000–500 cm^−1^, with a resolution of 4 cm^−1^ [33]. The resulting spectra were analyzed to identify functional groups and evaluate structural changes in lignocellulosic components.

### 2.13. Analysis Method of Contact Angle

A portion of the sieved sample was pressed into tablets under a pressure of 15 MPa for 1 min using an infrared tablet press. Each pressed sample was suspended between two glass slides positioned beneath the needle of the contact angle goniometer (JCY-3, Shanghai Fangrui Instrument Co., Ltd., Shanghai, China). A water droplet was placed on the tablet surface, and its spreading and penetration behavior was recorded. Images were captured every 2 s, with a total of 10 consecutive frames collected to analyze the dynamic wetting characteristics of the sample [34].

### 2.14. Statistical Analysis

Data were analyzed using SPSS Statistics (version 22.0; IBM Corporation, Armonk, NY, USA). After confirming normality (Shapiro-Wilk test) and homogeneity of variance (Levene’s test), one-way ANOVA was applied to examine group differences. For significant ANOVA results (*p* < 0.05), Duncan’s multiple range test was used for pairwise comparisons. Values were reported as means ± SEM, with statistical significance set at *p* < 0.05.

## 3. Results and Discussion

### 3.1. The Effect of the Concentration of NaOH on the Components and the Enzymatic Hydrolysis Performance of Corn Stover Pretreated by Low-Alkali Assisted ICSE

In this study, low-alkali assisted ICSE technology was employed to pretreat corn stover. Based on previous findings from our research group, NaOH was selected as the alkaline reagent due to its superior performance, the optimal conditions for ICSE of corn stover were steam pressure of 2.0 MPa and retention time of 360 s. A series of pretreatments using 1–5 wt% NaOH were conducted to systematically examine the changes in the composition of corn stover and evaluate the enzymatic hydrolysis efficiency under different alkali concentrations.

The overarching aim of the present study was to delineate the inhibitory role of lignin and its derivatives in constraining the enzymatic hydrolysis of corn stover. To achieve this, washed and unwashed solids were systematically employed as contrasting substrates. The washing procedure was intentionally incorporated as an experimental control to eliminate soluble pretreatment-derived byproducts, including low-molecular-weight lignin oligomers (water-soluble lignin) and lignin-derived aromatic aldehydes, both of which have been extensively reported to exert adverse effects on cellulase activity and stability. This comparison enabled a mechanistic distinction between inhibitor-derived effects and the intrinsic structural accessibility of pretreated biomass, providing the basis for interpreting glucose yield variations under different alkali concentrations.

The contents of cellulose, hemicellulose, and lignin in the control group (untreated corn stover) were 34.27%, 19.41%, and 28.51%, respectively, which are generally consistent with the corn stover composition reported by Lu et al. (cellulose: 37.67%, hemicellulose: 20.58%, lignin: 26.06%) [35]. As shown in Table 1, compared with the raw corn stover, the relative cellulose content increased across all pretreatment groups, while hemicellulose and lignin contents decreased. For instance, ICSE without any alkali increased cellulose content by 3.4% while reducing hemicellulose and lignin by 1.64% and 1.04%, respectively. This indicates that ICSE alone can physically disrupt the lignocellulosic matrix, resulting in partial dissolution of hemicellulose and lignin and a relative increase in cellulose content. With the addition of NaOH, the cellulose content continued to rise while lignin content declined, as higher alkali concentrations promoted cleavage of β–O–4 ether bonds, ester linkages, and some C–C bonds within the lignin structure. Notably, at 4% and 5% NaOH concentrations, the washed samples showed more favorable compositional changes. Furthermore, the combination of chemical disruption by alkali and physical effects from ICSE enhanced lignin solubilization and partial hemicellulose removal, leading to improved delignification and greater cellulose exposure.

Additionally, part of the pretreated corn stover was subjected to water washing prior to drying. Results showed that the washed samples consistently exhibited higher cellulose content than the unwashed groups. As shown in Table 1, in the absence of alkali, ICSE increased the cellulose content by only 1.18% in unwashed samples, compared to a 3.4% increase in the washed group. When 4% NaOH was used, the cellulose content in the washed sample was 5.88% higher than in the unwashed counterpart, while hemicellulose and lignin contents were lower by 2.31% and 1.69%, respectively. The observed differences are mainly attributable to water washing process, removing soluble components dominated by degraded lignin and its derivatives. Water washing reduces the retention of soluble lignin-derived substances during drying, thereby improving the accuracy of compositional analysis. In contrast, unwashed samples are prone to lignin redeposition on fiber surfaces, which artificially elevates lignin content and lowers cellulose levels. Consequently, washed samples more faithfully represent the structural changes induced by pretreatment. Since enzymatic hydrolysis is the central step in biomass conversion, enabling the depolymerization of polysaccharides into fermentable monosaccharides that provide essential carbon sources for high-value chemicals, these compositional and structural differences are expected to have a direct impact on hydrolysis performance.

**Figure 1:**
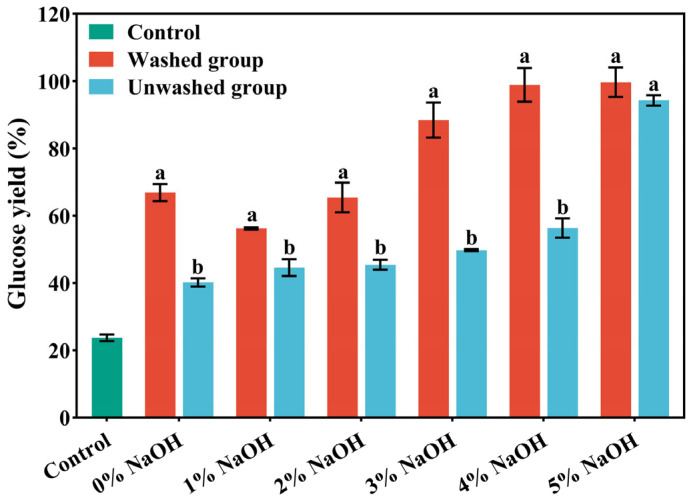
The glucose yield after enzymatic hydrolysis of corn stover pretreated with different concentrations of NaOH assisted instant catapult steam explosion for 72 h. Data are shown as mean ± SD (n = 3 per experimental group). Different superscripts within a column indicate significant differences (*p* < 0.05).

As shown in Figure 1, the removal of lignin during pretreatment markedly reduced non-productive cellulase adsorption and the physical shielding effect, thereby enhancing enzymatic accessibility. As the NaOH concentration increased from 1% to 5%, glucose yield followed a non-linear upward trend, first rising rapidly and then approaching a plateau, which suggests that 4–5% NaOH represents an optimal range balancing cost and performance. After 72 h of enzymatic hydrolysis, the glucose yields of the 4% NaOH washed group and the 5% NaOH washed group both exceeded 98%, surpassing the other pretreatment groups. This result indicates that under the combined influence of alkaline conditions and the high temperature and pressure of ICSE, part of the hemicellulose was depolymerized, cellulose microfibrils were fully exposed, the cellulose wrapping was weakened, and the synergistic effect of cellulase was promoted. In addition, water washing removed phenolic and aldehyde compounds attached to the material surface and further loosened the compact fiber structure through a swelling effect, which increased porosity, facilitated enzyme-substrate contact, and ultimately improved enzymatic hydrolysis efficiency.

### 3.2. Washed vs. Unwashed at 4% NaOH Assisted ICSE: Evidence for Lignin-Derived Water-Soluble Anti-Enzymatic Factors

As shown in Figure 1, under 4% NaOH assisted ICSE, the glucose yield of the washed sample reached 98.88%, whereas that of the unwashed counterpart was only 56.34%. This striking difference demonstrates that water washing effectively removes lignin-derived soluble inhibitors, enabling the hydrolysis efficiency of washed samples to approach the theoretical maximum. The results provide direct evidence that the reduced enzymatic hydrolysis in unwashed samples is primarily attributable to both soluble and insoluble lignin-derived factors rather than compositional variations alone. Accordingly, the subsequent identification and validation of water-soluble inhibitors were conducted using the washing solution obtained from the 4% NaOH pretreatment group.

**Figure 2:**
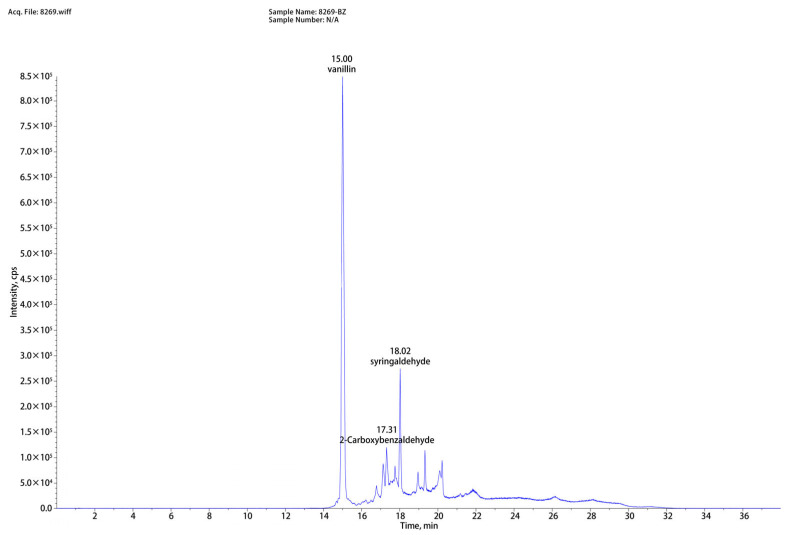
Representative mass spectra and chromatograms of corn straw hydrolysate pretreated by 4%NaOH assisted ICSE.

As shown in Figure 2, based on the liquid chromatography-mass spectrometry (LC-MS) analysis of washing solution, eight compounds (vanillin, syringaldehyde, 2-Carboxybenzaldehyde, lotusine, 3′-hydroxygenkwanin, anileridine, isosinensetin, N6-methyladenosine) were successfully identified. Among them, vanillin, syringaldehyde, and 2-carboxybenzaldehyde exhibited the highest peak intensities and were closely associated with lignin degradation. The disruption of lignin structure during low-alkali assisted ICSE explains their formation. Lignin consists of *p*-hydroxyphenyl, syringyl, and guaiacyl units; under alkaline and high-temperature/high-pressure conditions, cleavage of β–O–4 linkages generates aldehydes. Specifically, vanillin originates from the decomposition of guaiacyl units, syringaldehyde results from side-chain cleavage and oxidation of the dimethoxy groups on sinapyl alcohol, and 2-carboxybenzaldehyde is formed via side-chain cleavage and dehydrogenation of *p*-coumarol. By supplementing these compounds at concentrations corresponding to those measured in the washing solution and evaluating their effects on enzymatic hydrolysis, direct evidence can be obtained to elucidate the role of lignin-derived inhibitors in the overall anti-enzymatic mechanism.

To validate the inhibitory roles of the three major lignin-derived compounds, vanillin, syringaldehyde, and 2-carboxybenzaldehyde, they were reintroduced into the enzymatic hydrolysis system of washed samples pretreated with 4% NaOH assisted ICSE at concentrations corresponding to those detected in the washing solution. (Quantitative analysis via high-performance liquid chromatography showed that the concentrations of the inhibitors were 0.72 mg/mL for vanillin, 0.27 mg/mL for syringaldehyde, and 0.24 mg/mL for 2-carboxybenzaldehyde.) To account for possible variations and to explore the dose-response relationship, a gradient covering both the detected concentrations and higher levels was established. This design not only reflects the practical concentrations present in the hydrolysate environment but also provides a means to identify threshold effects beyond which enzymatic inhibition becomes significant.

**Figure 3:**
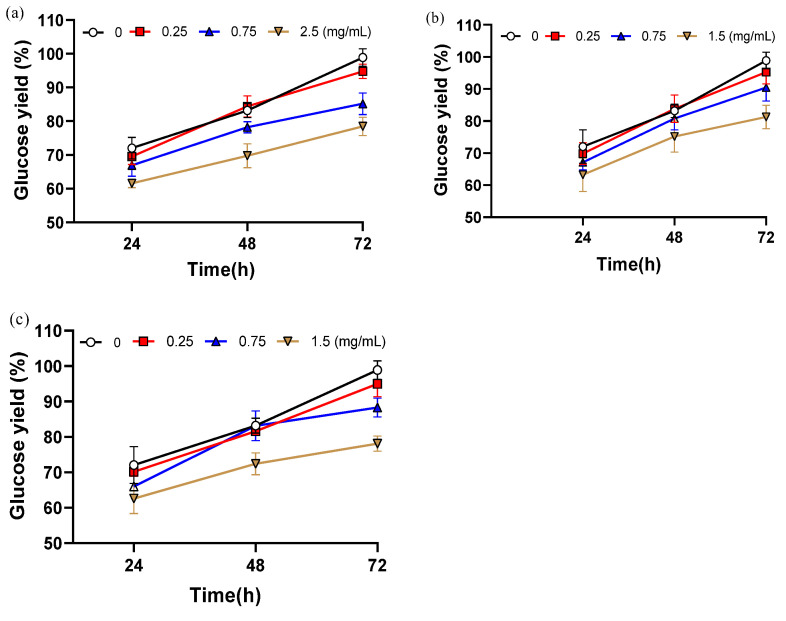
The glucose yield after adding different contents of inhibitors. (**a**) Glucose yield adding different contents of vanillin; (**b**) Glucose yield adding different contents of 2-carboxybenzaldehyde; (**c**) Glucose yield adding different contents of syringaldehyde. Data are shown as mean ± SD (n = 3 per experimental group).

As shown in Figure 3, the addition of these compounds consistently reduced hydrolysis efficiency compared with the control, and the extent of inhibition increased with concentration, confirming their significant negative impact on cellulase activity. All three compounds exhibited clear concentration-dependent inhibition. After 72 h of hydrolysis, glucose yield decreased by 20.4% with 2.5 mg/mL vanillin, by 20.8% with 1.5 mg/mL syringaldehyde, and by 17.6% with 1.5 mg/mL 2-carboxybenzaldehyde relative to the inhibitor-free control. The inhibitory effects are likely linked to structural features: the methoxy group (–OCH_3_) of vanillin may enhance hydrophobic interactions with the enzyme, while the ortho-hydroxyl group of syringaldehyde may contribute to steric hindrance.

When the concentration was as low as 0.25 mg/mL, glucose yields were only slightly reduced compared with the control, by 4.1% with vanillin, 3.9% with syringaldehyde, and 3.3% with 2-carboxybenzaldehyde. These results demonstrate a clear threshold effect in the inhibition of enzymatic hydrolysis by phenolic aldehydes. Under 5% NaOH assisted ICSE, the inhibitors generated are smaller in molecular weight and more volatile, allowing partial removal during the explosion process. In addition, higher alkali concentrations promote side-chain oxidation of lignin, converting phenolic aldehydes into carboxylic acids with weaker inhibitory effects. Together, these effects ensure that the actual concentration of phenolic inhibitors in the 5% NaOH unwashed samples remains below the critical threshold (<0.25 mg/mL), thereby maintaining glucose yields above 94%. While soluble phenolic aldehydes clearly play an important role, the substantial improvement observed in washed samples also suggests that water-insoluble lignin residues contribute to enzymatic inhibition.

### 3.3. Role of Alkali-Soluble Lignin as a Water-Insoluble Anti-Enzymatic Factor

To evaluate the contribution of residual lignin fractions, attention was directed to the portion of lignin retained in the pretreated solids but extractable under alkaline conditions. Although commonly termed alkali-soluble lignin, this fraction essentially represents part of the water-insoluble anti-enzymatic factors, in contrast to the water-soluble inhibitors directly dissolved in the pretreatment liquor. The inhibitory effect of this fraction was examined by extracting alkali-soluble lignin from unwashed samples and reintroducing it into the enzymatic hydrolysis system of washed samples, thereby simulating the inhibitory role of lignin fragments that persist in the solid matrix after pretreatment.

During low-alkali assisted ICSE pretreatment, part of the lignin dissolves into the liquid phase, whereas another fraction remains bound within the solid residue. Alkali extraction of both washed and unwashed materials enabled a comparison of the extractable lignin fractions and their relationship with hydrolysis performance. The extraction yield from the 4% NaOH unwashed material reached 43.28%, which was 1.36 times higher than that of the corresponding washed material (31.93%). This difference is likely associated with the combined effect of ICSE-induced shear forces and alkaline cleavage of β–O–4 ether linkages, which promote the release of covalently bound lignin. In contrast, water washing removes part of the soluble lignin fragments beforehand, resulting in a relatively lower extraction yield.

To further investigate the inhibitory effect of this fraction, the alkali-soluble lignin extracted from 4% NaOH unwashed material was reintroduced into the enzymatic hydrolysis system of washed samples. The hydrolysis system contained 0.5 g of substrate, with a lignin content of approximately 20%, corresponding to 0.1 g of lignin. Accordingly, the reintroduction range of alkali-soluble lignin was set between 0 and 0.1 g. As showed in Table 2, the results demonstrated a dose-dependent reduction in glucose yield. In the absence of added lignin, hydrolysis efficiency reached 98.88%. Supplementation with 0.05 g of alkali-soluble lignin reduced the yield to 93.71%, and further increases to 0.075 g and 0.1 g resulted in yields of 91.17% and 89.74%, respectively. These findings indicate that alkali-extractable lignin functions as a major water-insoluble anti-enzymatic factor when present above a threshold level. The reduced hydrolysis efficiency can be explained by a combination of non-productive adsorption of cellulases onto lignin surfaces, steric hindrance from lignin redeposition on cellulose, and the presence of phenolic derivatives that impair enzymatic activity.

Taken together, these results suggest that enzymatic inhibition originates not only from water-soluble compounds but also from residual lignin fractions embedded in the solid phase. To clarify how changes in alkali concentration influence the structural characteristics of such lignin and their inhibitory effects, subsequent analyses focused on comparing the 4% unwashed and 5% unwashed samples. This comparison provided further insight into how lignin solubilization, condensation, and redistribution at different alkali levels govern enzymatic hydrolysis efficiency.

### 3.4. Impact of Water-Soluble Lignin on Enzymatic Hydrolysis Performance Under Varying Alkali Severities

Efficient enzymatic hydrolysis is strongly influenced by the relative contributions of soluble inhibitors and insoluble lignin residues. Water washing can partly remove soluble inhibitors and thereby improve digestibility, but this operation consumes large amounts of water, generates wastewater that is difficult to treat, and increases process costs. A more sustainable approach is to identify pretreatment conditions that intrinsically mitigate inhibitory effects without relying on washing. Within the low-alkali assisted ICSE framework, changes in alkali severity are expected to alter lignin solubilization, condensation, and redistribution, which in turn affect the balance between soluble and insoluble fractions. Thus, comparing 4% and 5% NaOH pretreatments under unwashed conditions provides a practical means to disentangle the role of alkali concentration from washing effects and to directly evaluate how lignin partitioning relates to hydrolysis efficiency.

As summarized in Table 3, clear differences emerged between the two pretreatment severities. The 4% NaOH unwashed material contained 21.14 mg/g water-soluble lignin, exhibited an alkali-soluble lignin yield of 43.28%, and achieved a glucose yield of only 56.34%. In contrast, the 5% NaOH unwashed material showed a higher content of water-soluble lignin (32.09 mg/g) but a lower alkali-soluble lignin yield (38.22%), and reached a glucose yield of 94.26%. These findings indicate that increasing alkali concentration not only shifts lignin from the insoluble to the soluble fraction but also exerts a decisive influence on hydrolysis performance. At 4% NaOH, limited delignification and the persistence of insoluble lignin explain the low glucose yield. At 5% NaOH, more lignin was released into the liquid phase, reducing the proportion of insoluble residues and thereby alleviating steric hindrance and non-productive enzyme binding. Although the 5% condition generated a higher overall content of soluble lignin, the concentrations of phenolic aldehydes remained below the inhibitory threshold, allowing glucose yields to remain above 94%.

Overall, this comparative analysis highlights that enzymatic hydrolysis efficiency under low-alkali ICSE is not determined solely by soluble inhibitors, but is critically shaped by the quantity and structural state of insoluble lignin within the pretreated material.

### 3.5. Mechanism Analysis of Improving Enzymatic Hydrolysis Efficiency by Low-Alkali Assisted ICSE

The reintroduction experiments demonstrated that alkali-extractable lignin acts as a representative water-insoluble anti-enzymatic factor, exerting a dose-dependent inhibitory effect on cellulose saccharification. These findings clarified the functional roles of soluble and insoluble fractions, but further mechanistic insight requires examination of how alkali severity alters lignin structure and redistribution during ICSE. Although water washing can improve hydrolysis efficiency, it is accompanied by drawbacks such as high water consumption, wastewater treatment challenges, and additional cost. Therefore, attention was directed to unwashed materials, where the contrast between 4% and 5% NaOH pretreatment provided a practical framework to isolate the effect of alkali concentration. Notably, the 5% unwashed material maintained glucose yields above 94% despite the absence of washing, whereas the 4% condition exhibited reduced conversion, suggesting that differences in lignin solubilization and redeposition underlie the observed performance gap.

To elucidate these mechanisms, both soluble lignin-derived compounds and solid-phase lignin residues were analyzed. Quantitative measurements of soluble inhibitors were combined with structural and interfacial characterizations of the materials using scanning electron microscopy (SEM), Fourier transform infrared spectroscopy (FT-IR), X-ray diffraction (XRD), and contact angle analysis, aiming to clarify how lignin transformations at different alkali loadings influence enzymatic accessibility.

#### 3.5.1. Influence of Water-Soluble Lignin on Enzymatic Hydrolysis Efficiency

As shown in Table 3, during the low-alkali assisted ICSE pretreatment process, when the NaOH concentration increased from 4% to 5%, the glucose yield increased from 56.34% to 94.26%. The increase in NaOH concentration directly affects the degradation of lignin, intensifies the breakage of the β–O–4 ether bonds in lignin, and promotes the release of more lignin into the pretreatment system in the form of free water-soluble fragments. By measuring the content of water-soluble lignin in the pretreatment system, the results show that the content of water-soluble lignin in the unwashed materials pretreated by 5% NaOH assisted ICSE is 32.09 mg/g of raw material, which is 51.80% higher than that under the 4% NaOH condition. Meanwhile, the yield of alkali-soluble lignin in the raw material pretreated by 5% NaOH assisted ICSE is 38.22%, which is lower compared with that under the 4% NaOH condition. These results indicate that with the increase of NaOH concentration, more lignin is degraded into water-soluble lignin. Some studies have shown that water-soluble lignin reversibly binds to the adsorption domain of cellulase to form a lignin-cellulase complex, effectively reducing the non-productive adsorption of cellulase by the lignin, thereby improving the enzymatic hydrolysis conversion efficiency of biomass [36].

The above results indicate that moderately increasing the alkali concentration can more thoroughly degrade and dissolve lignin. This verifies the regulatory effect of lignin structural transformation on enzymatic hydrolysis, and reveals the crucial role of optimizing pretreatment parameters in balancing the generation of inhibitors and protecting enzyme activity. It provides theoretical support for the efficient conversion of lignocellulose.

#### 3.5.2. Analysis of Surface Morphology

**Figure 4:**
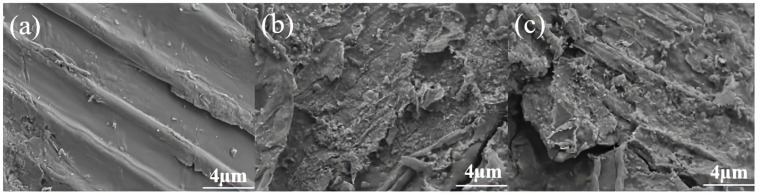
Scanning electron microscopy images of corn stover pretreated with low alkali assisted instant catapult steam explosion. (**a**) represents the untreated corn stover; (**b**) represents the corn stover in the group with 4% NaOH assisted instant catapult steam explosion and without washing; (**c**) represents the corn stover in the group with 5% NaOH assisted instant catapult steam explosion and without washing.

SEM analysis is commonly used to study the changes in the microstructure of biomass during the pretreatment process, the results are showed in Figure 4. Similar to the study by Xie et al. [37], the untreated corn stover is arranged closely and regularly as a whole, presenting a complete and orderly fiber structure. Its surface is relatively smooth without obvious fiber structure [38]. For the pretreated corn stover, due to the mechanical force generated by the instantaneous pressure relief, the secondary wall S2 layer is peeled off, the original biomass framework collapses, and the pit membranes rupture to form microporous channels, which increases the surface roughness. The lignocellulose structure is damaged, part of the lignin and hemicellulose are removed, and the cellulose is fully exposed, thus improving the enzymatic accessibility of cellulose.

As shown in Figure 4c, the surface of the corn stover after being treated with 5% NaOH without water washing is rougher and has a larger specific surface area. The corn stover pretreated with 5% NaOH exhibits a more intense fiber swelling effect, which leads to a more thorough dissociation of the microfibril bundles and exposes more layered structures. Moreover, 5% NaOH causes a more complete dissociation of the lignin-carbohydrate complex (LCC), forming a denser microporous structure during ICSE. Most importantly, after pretreatment with 5% NaOH, more oligosaccharides produced by the degradation of hemicellulose are retained on the fiber surface. These hydrophilic substances shrink during the drying process to form nanoscale wrinkles. This enhanced rough surface provides more anchoring sites for cellulase, increasing the amount of enzyme adsorption. This is also a key morphological factor contributing to the high enzymatic hydrolysis efficiency of the 5% NaOH unwashed sample.

#### 3.5.3. Analysis of X-Ray Diffraction

**Figure 5:**
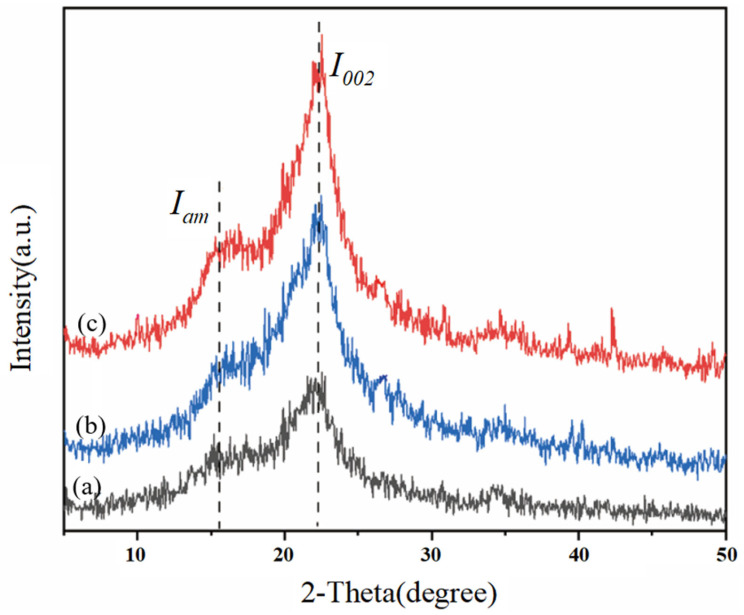
XRD pattern of corn stover pretreated with low alkali assisted instant catapult steam explosion. (a) represents the untreated corn stover; (b) represents the corn stover in the group with 4% NaOH assisted instant catapult steam explosion and without washing; (c) represents the corn stover in the group with 5% NaOH assisted instant catapult steam explosion and without washing.

Crystallinity is one of the factors indicating the pretreatment effect of biomass and is related to the enzymatic hydrolysis efficiency during the enzymatic hydrolysis process [39]. To evaluate the influence of low-alkali assisted ICSE pretreatment on the crystallinity (CrI) of the materials treated with 4% NaOH without water washing and 5% NaOH without water washing, XRD technology was used to determine the CrI values of the cellulose-rich parts. As shown in Figure 5, the samples have obvious characteristic diffraction peaks of cellulose at diffraction angles of 16.15° and 22.3°, presenting a typical cellulose I structure. Compared with the control group, the peak intensity of the pretreated corn stover is significantly enhanced. When comparing the unwashed corn stover pretreated with 4% NaOH and 5% NaOH assisted ICSE, the peak surface of the (002) crystal plane of the corn stover pretreated with 5% NaOH is relatively narrower and clearer, but there is no obvious change in the diffraction peak pattern of the samples, indicating that only the intensity of the characteristic peaks is affected. As shown in Table 4, the crystallinity and crystallinity index of the corn stover treated with 5% NaOH are increased by 3.24% and 1.87% respectively compared with the group treated with 4% NaOH, and are significantly higher than those of the untreated raw material. This is because 5% NaOH has a stronger swelling effect, which can preferentially dissolve the amorphous region with low degree of polymerization and at the same time promote the rearrangement of residual cellulose molecules to form a more regular Type I structure. This “selective purification” process forms a unique “high-crystallinity-multiple-defect” structure: on one hand, the improved crystallization integrity provides a directional unzipping template for cellulase; on the other hand, ICSE induces the generation of nanoscale cracks in the crystalline region, increasing the effective accessible surface area. More importantly, 5% NaOH reduces the surface coverage of lignin, and its degradation products exist more in the form of low molecular weight. These characteristics work together to retain the guiding advantage of the crystalline region for the enzyme.

#### 3.5.4. Analysis of Infrared Spectroscopy

**Figure 6:**
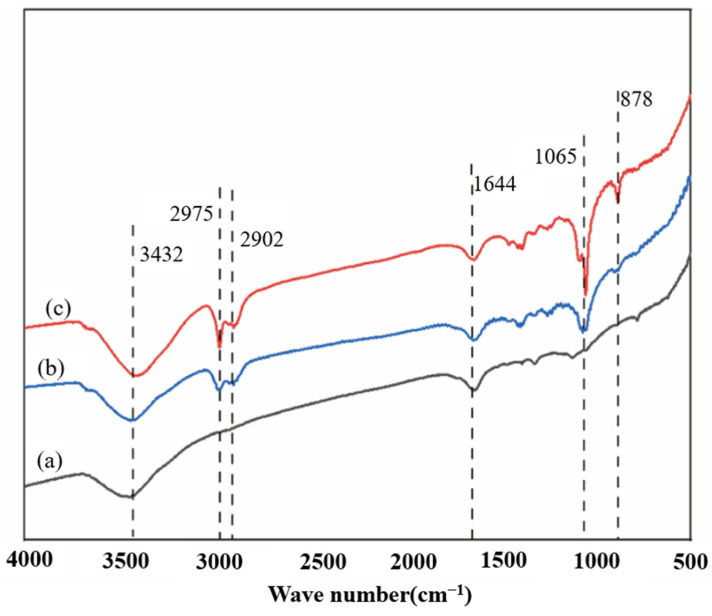
Infrared spectra of corn stover after pretreatment with low-alkali assisted instant catapult steam explosion. (a) represents the untreated corn stover; (b) represents the corn stover in the group with 4% NaOH assisted instant catapult steam explosion and without washing; (c) represents the corn stover in the group with 5% NaOH assisted instant catapult steam explosion and without washing.

To further study the changes in the chemical structure of corn stover before and after water washing, FT-IR analysis was carried out on the unwashed corn stover after explosion within the range of 4000–500 cm^−1^. As shown in Figure 6, the infrared spectroscopy analysis indicates that the pretreatment of unwashed corn stover with 5% NaOH assisted ICSE shows significant advantages over that with 4% NaOH, which directly explains the reason for its higher enzymatic hydrolysis efficiency. At 3432 cm^−1^, the intensity of the O–H stretching vibration peak in the 5% NaOH group is enhanced, and the peak width increases, confirming that the cellulose is more fully swollen, exposing more sites for enzyme action [40]. The 17 cm^−1^ red shift of the peak at 2902 cm^−1^ indicates that the oxidation of the lignin side chain is more thorough and the degradation of lignin is more complete [41]. The increase in the intensity of the peak at 1065 cm^−1^ in the polysaccharide region is consistent with the XRD results, showing that the order degree of the cellulose crystalline region is improved, providing a directional template for enzymatic hydrolysis [42].

The characteristic peak of soluble lignin and its derivatives was detected at 1605 cm^−1^ in the samples. At this wavelength, the intensity of the vibration peak in the 5% NaOH group is significantly enhanced, indicating that the cleavage of β–O–4 ether bonds in lignin generates more small molecular fragments (such as dimers and trimers), and the soluble lignin exists in the form of low molecular weight fragments. The cleavage of ether bonds and the exposure of phenolic hydroxyl groups are the main factors for the change in the peak shape. The appearance of a small peak at the wavelength of 878 cm^−1^ indicates that under these pretreatment conditions, local changes in specific chemical bonds (such as β-glycosidic bonds or lignin fragments) in corn stover are induced, further verifying the changes in crystallinity and the structure of lignin. Ultimately, it is demonstrated that the 5% NaOH group, while maintaining high crystallinity, has an increased enzyme-accessible surface area, ultimately achieving a higher enzymatic hydrolysis efficiency.

#### 3.5.5. Surface Hydrophilicity and Interfacial Behavior by Contact Angle

**Figure 7:**
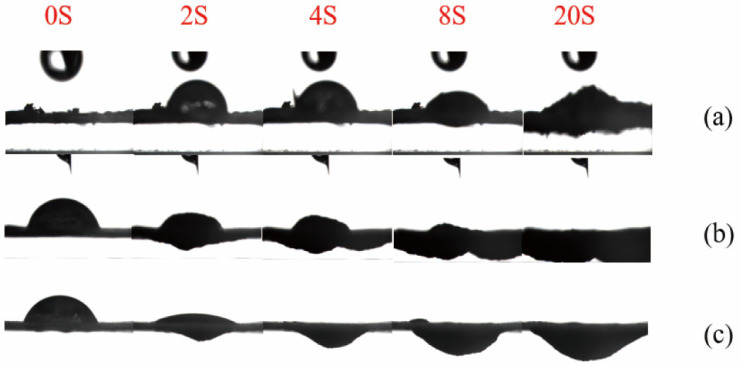
Contact angle diagrams of corn stover after pretreatment with low-alkali assisted instant catapult steam explosion. (**a**) represents the untreated corn stover; (**b**) represents the corn stover in the group with 4% NaOH assisted instant catapult steam explosion and without washing; (**c**) represents the corn stover in the group with 5% NaOH assisted instant catapult steam explosion and without washing.

The contact angle tester captures an image every 2 s. Images of the liquid droplets falling on the samples at different time points are intercepted to observe the entire penetration process of the liquid droplets on the samples. On the same time axis, the untreated corn stover, the unwashed sample of corn stover after 4% NaOH assisted ICSE, and the unwashed sample of corn stover after 5% NaOH assisted ICSE are selected. During the contact angle test process, five images at 0 s, 2 s, 4 s, 10 s, and 20 s are respectively selected to form a set of photos. As shown in Figure 7, for the 5% NaOH assisted ICSE group, the diffusion speed of the liquid droplets after falling is faster than that of the untreated corn stover. Moreover, under the action of gravity, the penetration speed of the pretreated sample is faster than that of the untreated sample.

The 5% NaOH group has already expanded at 2 s, and the liquid droplets have completely penetrated at 4 s. It is speculated that this is caused by three aspects. Firstly, the micron-sized pores generated by ICSE and the nanoscale soluble lignin fragments jointly construct a hierarchical penetration channel. Secondly, the carboxylate (–COO^−^) formed by the oxidation of lignin has strong hydration ability, which rapidly causes fiber expansion within 2 s. Thirdly, the pretreatment of ICSE combined with 5% NaOH removes lignin more thoroughly and exposes a large number of hydrophilic groups. These hydrophilic functional groups increase the surface polarity and decrease the interfacial energy barrier between lignin and the aqueous phase, thereby facilitating liquid penetration and enzyme-substrate interactions. The enhanced interfacial hydrophilicity improves the effective adsorption and catalytic efficiency of cellulase, ultimately leading to higher enzymatic hydrolysis efficiency.

These results of this section indicate that the improvement in enzymatic hydrolysis efficiency under 5% NaOH assisted ICSE primarily originates from the structural rearrangement and redistribution of lignin, which weaken the non-productive adsorption of cellulase on hydrophobic lignin surfaces. The oxidative cleavage of β–O–4 linkages and formation of polar oxygen-containing groups promote interfacial hydrophilic reconstruction, as reflected by the reduced contact angle and enhanced enzyme accessibility. This transformation from a condensed hydrophobic barrier to a hydrophilic, enzyme-compatible interface provides the molecular basis for the superior digestibility of 5% NaOH assisted ICSE pretreated corn stover.

**Figure 8:**
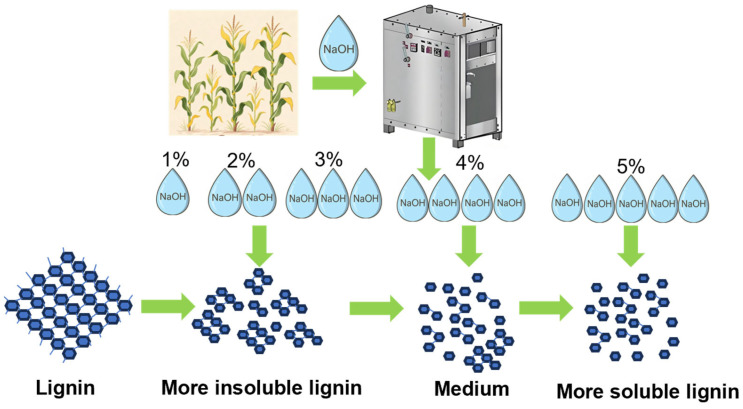
Schematic illustration of lignin structural rearrangement pathway under low-alkali assisted ICSE pretreatment.

To provide an integrated view of the inhibitory mechanisms, a summarizing schematic was constructed (Figure 8). In unwashed materials subjected to low-alkali assisted ICSE, the predominant mode of inhibition is strongly governed by alkali severity. At comparatively low NaOH concentrations, the extent of structural disintegration remains insufficient to solubilize lignin to a meaningful degree, resulting in the majority of lignin persisting as water-insoluble residues intimately associated with the fiber surface. Under these conditions, inhibition is dominated by the solid-phase lignin, where steric obstruction and non-productive adsorption substantially restrict enzyme accessibility. As the alkali concentration increases, lignin undergoes progressively more extensive structural rearrangement-marked by β–O–4 bond cleavage, oxidative modification of side chains, and the emergence of additional polar functionalities-which collectively enhance its solubility and promote the release of lignin fragments into the liquid phase. This shift reduces the quantity of insoluble lignin retained within the solid matrix. At 5% NaOH, although the generation of soluble phenolic inhibitors is elevated, the concomitant decline in insoluble lignin, together with the relatively reversible interactions between enzymes and soluble lignin fragments, markedly mitigates the non-productive adsorption associated with the solid lignin phase. The net inhibitory intensity is therefore diminished, resulting in significantly improved enzymatic digestibility compared with lower-alkali pretreatment conditions.

## 4. Conclusions

A low-alkali assisted ICSE pretreatment technology is demonstrated as an effective strategy to enhance the enzymatic hydrolysis of corn stover. The 5% NaOH concentration yielded the highest glucose conversion, with washed and unwashed groups reaching 99.66% and 94.26%, respectively. Three water-soluble anti-enzymatic factors in the washing solution were identified and verified, exhibiting concentration-dependent inhibitory effects. Alkali-soluble lignin extracted from unwashed samples significantly reduced glucose yield when backfilled, confirming its role as a water-insoluble anti-enzymatic factor. Moreover, the pretreatment induced lignin structural rearrangement and redistribution, leading to partial oxidation of β–O–4 linkages and generation of polar oxygen-containing groups. This transformation altered lignin solubility and interfacial properties, enabling a more hydrophilic and enzyme-accessible substrate surface. Additionally, the higher content of water-soluble lignin in the 5% NaOH pretreatment group may competitively bind cellulase and mitigate its non-productive adsorption to water-insoluble lignin, thereby improving hydrolysis efficiency. Structural characterizations using SEM, XRD, FT-IR, and contact angle analysis further revealed enhanced fiber porosity, cellulose crystallinity, and surface hydrophilicity. These results collectively demonstrate that lignin structural rearrangement serves as a central mechanism for alleviating lignin-derived inhibition, providing mechanistic insights and practical guidance for optimizing lignocellulosic biomass pretreatment and advancing efficient, scalable, and sustainable biorefinery processes.

## Figures and Tables

**Table 1:** The content of corn stover pretreated by different concentrations of NaOH assisted instant catapult steam explosion (2.0 MPa-360 s).

Preprocessing Conditions	Cellulose (%)	Hemicellulose (%)	Lignin (%)
Raw corn stover	34.27 ± 0.60	19.41 ± 0.29	28.51 ± 0.17
Control (washed)	37.67 ± 0.13	17.77 ± 0.58	27.47 ± 0.87
1% NaOH (washed)	39.67 ± 0.15	15.02 ± 0.15	25.70 ± 0.47
2% NaOH (washed)	39.89 ± 0.47	16.38 ± 0.11	24.75 ± 1.70
3% NaOH (washed)	40.21 ± 0.05	15.47 ± 0.69	22.70 ± 0.52
4% NaOH (washed)	41.99 ± 0.99	15.39 ± 0.29	20.69 ± 0.06
5% NaOH (washed)	40.42 ± 0.61	15.03 ± 0.04	20.11 ± 1.17
Control (unwashed)	35.45 ± 0.60	17.03 ± 0.11	28.27 ± 1.76
1% NaOH (unwashed)	35.64 ± 0.26	16.38 ± 0.19	26.21 ± 0.91
2% NaOH (unwashed)	35.09 ± 0.42	16.81 ± 0.06	25.91 ± 0.66
3% NaOH (unwashed)	35.14 ± 0.72	17.46 ± 0.30	22.67 ± 0.52
4% NaOH (unwashed)	36.11 ± 0.33	17.70 ± 0.21	22.38 ± 0.63
5% NaOH (unwashed)	35.52 ± 2.56	17.08 ± 1.73	22.18 ± 0.19

Data are shown as mean ± SD (n = 3 per experimental group).

**Table 2:** Enzymatic hydrolysis efficiency after adding different amounts of alkali-soluble Lignin solid.

Addition Amount of Alkali-Soluble Lignin Solid (g)	Glucose Yield (%)
0	98.88 ± 4.97
0.05	93.71 ± 3.75
0.075	91.17 ± 2.88
0.1	89.74 ± 3.14

The volume of the enzymatic hydrolysis system is 20 mL. Data are shown as mean ± SD (n = 3 per experimental group).

**Table 3:** Contents of water-soluble lignin and yields of alkali-soluble lignin under different pretreatment conditions.

Pretreatment System	Content of Water-Soluble Lignin (mg/g)	Yield of Alkali-Soluble Lignin (%)	Glucose Yield (%)
4% NaOH (unwashed)	21.14 ± 0.84	43.28 ± 0.97	56.34 ± 2.85
5% NaOH (unwashed)	32.09 ± 0.74	38.22 ± 1.62	94.26 ± 1.56

Data are shown as mean ± SD (n = 3 per experimental group).

**Table 4:** Crystallinity of unwashed corn straw after low alkali assisted instant catapult steam explosion pretreatment.

Pretreatment System	CrI (%)	Crystalline Portion (%)
Raw corn stover	33.65	58.62
4% NaOH (unwashed)	50.00	66.70
5% NaOH (unwashed)	53.24	68.57

## Data Availability

The additional data supporting the manuscript are available from the corresponding author upon request.
